# A microbial muramidase improves growth performance and reduces inflammatory cell infiltration in the intestine of broilers chickens under *Eimeria* and *Clostridium perfringens* challenge

**DOI:** 10.1016/j.psj.2023.103226

**Published:** 2023-11-02

**Authors:** Cristiano Bortoluzzi, Leticia C. Bittencourt, Estefania Perez-Calvo, Bruna L. Belote, Igor Soares, Elizabeth Santin, José Otávio Berti Sorbara, Luiz F. Caron

**Affiliations:** ⁎ DSM-Firmenich, Kaiseraugst, Switzerland; † DSM-Firmenich, São Paulo, Brazil; ‡ ISI Institute, Londrina, Paraná, Brazil; § Federal University of Paraná, Curitiba, Brazil

**Keywords:** carotenoids, intestinal permeability, muramidase, peptidoglycan, welfare

## Abstract

The objective of the present studies was to evaluate muramidase (**MUR**) supplementation in broilers under *Eimeria* and/or *Clostridium perfringens* challenge. For this, 2 experiments were conducted. Experiment 1. A total of 256 one-day old male Cobb 500 chicks were placed in battery cages in a completely randomized design, with 5 treatment groups, 7 replicate cages per treatment and 8 birds per cage. The treatments were: nonchallenged control (**NC**), challenged control (**CC**), CC + MUR at 25,000 or 35,000 LSU(F)/kg, and CC + Enramycin at 10 ppm (positive control—**PC**). Challenge consisted of 15× the recommended dose of coccidiosis vaccine at placement, and *Clostridium perfringens* (10^8^ CFU/bird) inoculation at 10, 11, and 12 d. Macro and microscopic evaluation, immunohistochemistry, and gene expression were evaluated at 7, 14, 21, and 28 d of age. Experiment 2. A total of 1,120 one-day old male Cobb 500 chicks were placed in floor pens with fresh litter in a completely randomized design, with 4 treatment groups, 8 replicate pens per treatment, and 35 birds per pen. The treatments were: Control, supplementation of MUR at 25,000 or 45,000 LSU(F)/kg, and a positive control (basal diet plus Enramycin). At 10, 11, and 12 d of the experiment all the birds were inoculated by oral gavage with a fresh broth culture of a field isolate *Clostridium perfringens* (0.5 mL containing 10^6^ CFU/bird). It was observed that in Experiment 1 MUR supplementation reduced the infiltration of macrophages and CD8+ lymphocytes in the liver and ileum of infected birds, downregulated IL-8 and upregulated IL-10 expression. In Experiment 2, MUR linearly improved the growth performance of the birds, increased breast meat yield, and improved absorption capacity. MUR supplementation elicited an anti-inflammatory response in birds undergoing a NE challenge model that may explain the improved growth performance of supplemented birds.

## INTRODUCTION

The dietary supplementation of muramidase (**MUR**) has shown to have positive impact on the growth performance of broiler chickens (Sais et al., 2020; Wang et al., 2021; Brugaletta et al., 2022; Goes et al., 2022). The MUR enzyme targets and specifically hydrolyzes peptidoglycans (**PGN**) that are present in the gastrointestinal tract (**GIT**) of animals, as part of the normal bacterial cell turnover (Vidal et al., 2005; Sytwala et al., 2015), rather than the antinutritional factors present in the feed. Since the PGN fragments in the GIT may be recognized by the intestinal immune cells and activate an inflammatory response (Kogut et al., 2018), it is possible to hypothesize that the dietary supplementation of MUR may provide an immunological advantage to the host in dealing with the stressor (PGN). The potential anti-inflammatory effect of this enzyme could, in fact, be translated into a nutrient sparing benefit (improved digestibility) as observed previously (Goodarzi Boroojeni et al., 2019; Goes et al., 2022; Perez-Calvo et al., 2023).

The lysis of PGN by MUR reduces inflammation, improves nutrient absorption, and redirects nutrients to animal growth (Amer et al., 2023), by 2 mechanisms, mainly: reduction of PGN itself, and modulation of inflammatory mediators release. This is mainly explained by the fact that the muropeptides, breakdown products of PGN, alter the inflammatory response in the GIT (Traub et al., 2006). Amer et al. (2023) have demonstrated a positive impact of MUR supplementation on the immune system of broiler chickens by the increase in blood concentration of globulins, lysozyme, IL-10, and complement 3. The significant increase of IL-10 by more than 2 folds, even with the lowest supplementation dose of MUR, is of paramount importance in demonstrating the attenuating inflammatory effect of MUR as IL-10 is well-known for its immune regulatory/anti-inflammatory effect that drives a tolerance state during chronic inflammatory conditions. Cardoso Dal Pont et al. (2023) hypothesized that in chickens, following the innate response, the adaptive immune response is characterized by a reduction of macrophage stimulatory cytokines, and increase in chronic inflammation-related cytokines, such as IL-16 and IL-10. Therefore, evaluations during and after the immune stimulus are essential to show how MUR works during the acute inflammatory response promoted by pathogens.

In the poultry industry worldwide, *Clostridium perfringens* has caused major economic losses as it may cause necrotic enteritis (**NE**), and coccidiosis infection has been considered one of the important predisposing factors of NE. It has been reported that birds supplemented with MUR under an *Eimeria* challenge showed higher plasmatic carotenoids, as indicator of good gastrointestinal functionality, compared to nonsupplemented animals and similar to Enramycin (Goes et al., 2022); however, no immune assessment has been performed under such challenge. We hypothesized that the supplementation of MUR mitigates the negative effects of *Eimeria* and *Clostridium perfringens* in broiler by attenuating the inflammatory process. The objective of the present studies was to evaluate different doses of supplementation of MUR in broilers under *Eimeria* and/or *Clostridium perfringens* challenge on the growth performance, intestinal histopathology and immunohistochemistry, expression of immune-related genes, blood carotenoids concentration, and carcass yield.

## MATERIALS AND METHODS

### Experiment 1

The experiment was conducted at the Center of Immune Responses in Poultry at the Federal University of Paraná. All the procedures followed the guidelines and were approved by the internal Animal Care Committee under number 081/2016.

#### Animals and Experimental Design

A total of 256 one-day old male Cobb 500 chicks were used in this trial. The birds were placed in battery cages in a completely randomized design. The experiment consisted of 5 treatment groups with 7 replicate cages per treatment and 8 birds per cage. Each cage contained sterilized litter, nipple drinkers, and automatic temperature control. The treatments were: nonchallenged control (**NC**), challenged control (**CC**), CC + supplementation of MUR at 25,000 LSU(F)/kg, CC + supplementation of MUR at 35,000 LSU(F)/kg, and CC + Enramycin at 10 ppm (positive control—**PC**). The MUR used in the present study is same used by Goes et al. (2022), and its activity and recovery rates are presented in Table 3.

The birds had *ad libitum* access to water and feed during the entire experimental period that lasted 28 d. The birds were allocated into different rooms with equal environmental conditions, such as negative pressure ventilation, light intensity, and geographical location inside the building. The NC birds were allocated in a separated room to avoid cross contamination. Feed in mash form was based on corn, soybean meal, and meat and bone meal to meet or exceed the requirements of the birds and following the specifications for each phase: Starter (0–14 d) and grower (14–28 d). All the diets contained phytase (RONOZYME HiPhos GT) at 1,000 FYT/kg of feed and 40 ppm of CAROPHLL yellow 10%. Anticoccidials were not included in the basal diets (Table 1).

**Table 1:** Composition and calculated analysis of the experimental feeds. Experiments 1 and 2.

Ingredients, %	Starter 1–14 d	Grower 14–28 d	Starter 1–21 d	Grower 22–35 d	Finisher 36–42 d
	Experiment 1		Experiment 2		
Corn	58.9	60.6	50.9	57.2	60.5
Soybean meal	33.4	32.3	41.7	34.2	30.4
Meat and bone meal	3.60	2.90	-	-	-
Salt	0.46	0.44	0.46	0.43	0.41
Choline Chloride	0.06	0.06	0.08	0.06	0.05
Soybean oil	1.55	2.00	3.90	5.40	6.50
Dicalcium phosphate	0.12	0.00	0.89	0.69	0.62
Limestone	0.78	0.68	1.30	1.21	1.01
DL-Methionine	0.385	0.320	0.296	0.272	0.186
L-Lysine	0.360	0.275	0.134	0.190	0.103
L-Threonine	0.140	0.088	0.068	0.090	0.032
Vitamin Premix^1^	0.150	0.150	0.150	0.120	0.100
Mineral Premix^2^	0.050	0.050	0.050	0.050	0.050
HiPhos GT	0.010	0.010	0.010	0.010	0.010
BHT	0.010	0.010	0.010	0.010	0.010
CAROPHYLL Yellow 10%	0.040	0.040	0.040	0.040	0.040
Monensin	-	-	0.055	0.000	0.000
Salinomycin	-	-	0.000	0.055	0.055
Total, kg	100	100	100	100	100
Calculated analysis					
AMEn, kcal/kg	3,000	3,053	3,000	3,180	3,300
Crude Protein, %	22.0	21.2	23.0	20.0	18.5
Calcium, %	1.00	0.85	0.95	0.85	0.75
Av. P , %	0.47	0.41	0.45	0.40	0.38
Lys dig, %	1.32	1.22	1.30	1.16	1.00
Met dig, %	0.67	0.60	0.61	0.55	0.45
Thr dig, %	0.86	0.79	0.87	0.79	0.68
Trp dig, %	0.23	0.22	0.27	0.23	0.21
Arg dig, %	1.36	1.31	1.50	1.28	1.17

1 Vit. A 9,000,000 UI/kg; Vit. D3 2,500,000 UI/kg; Vit. E 20,000 UI/kg; Vit. K3 2,500 mg/kg; Vit. B1 2,000 mg/kg; Vit. B2 6,000 mg/kg; pantothenic acid 12 g/kg; Vit. B6 3,000 mg/kg; Vit. B12 15,000 mcg/kg; nicotinic acid 35 g/kg; folic acid 1,500 mg/kg; biotin 100 mg/kg; selenium 250 mg per kg of premix.

2 Iron 100 g/kg; cooper 20 g/kg; manganese 130 g/kg; cobalt 2,000 mg/kg; zinc 130 mg/kg; iodine 2,000 mg per kg of premix.

#### Experimental Challenge

At the day of placement, the challenged groups were given, by oral gavage, 15× the recommended dose of coccidiosis vaccine (Bio-Coccivet R—Laboratório Biovet Brazilian Laboratory, *E. acervulina, E. brunetti, E. maxima, E. necatrix, E. praecox, E. tenella*, and *E. mitis*), and at 10, 11, and 12 d of the experiment challenged birds were inoculated by oral gavage with a fresh broth culture of a field isolate *Clostridium perfringens* (10^8^ CFU/bird; Belote et al., 2018).

#### Necropsy and Sampling

At 7, 14, 21 and 28 d of age 1 bird/replicate was euthanized for necropsy macroscopic evaluation, and samples collection.

#### ISI Macroscopic Evaluation

After euthanasia by cervical dislocation, the birds had their health status systematically evaluated through the “I See Inside” (**ISI**) methodology (Kraieski et al., 2017). Various aspects of the birds were macroscopically assessed, including locomotor, respiratory, intestinal (duodenum, jejunum, ileum, and cecum), and the gastrointestinal tract (liver, yolk, proventriculus, gizzard, and pancreas). These macroscopic evaluations were carried out through the scoring of parameters from 0 to 3, where 0 = no alteration; 1 = mild; 2 = moderate; and 3 = severe alteration. For each parameter, the applied score was multiplied by its respective impact factor, which ranged from 1 to 3, according to the negative impact of that alteration on the organ functionality (3 = highest impact). The products of these multiplications were summed up to generate an ISI Total Score for each animal, where a higher value indicates a poorer health status.

#### ISI Histopathological Evaluation

For the histopathological evaluation, samples of ileum and liver were collected during the necropsy and immediately fixed in formaldehyde 10% for at least 24 h. These samples were placed in a circulator and treated with different concentrations of alcohol and toluene. The blocks were then embedded into paraffin and cut in 3 µm slices which were stained with hematoxylin and eosin (**H&E**) and alcian blue. For histopathological analysis, a total of 20 villi per bird were evaluated in 10× magnification (using 40× magnification to confirm alterations) under optical microscope (Nikon Eclipse E200, Sao Paulo, Brazil). The ISI microscopic methodology (patent: INPI BR 1020150036019) was used to measure histologic alterations on the intestine (Belote et al., 2018), and a final intestinal health index was produced.

Similar to the macroscopic analysis, scores ranging from 0 to 3 (0 = no alteration; 3 = severe alteration) are assigned to a predefined parameters observed through optical microscopy in the samples of ileum and liver. The intestinal assessment involved the scoring of lamina propria and epithelial thickness, enterocytes proliferation, inflammatory cell infiltration in the epithelium and in the lamina propria, congestion, and the presence of *Eimeria* spp. oocysts in the intestinal mucosa. In the liver, the following items were scored: congestion, cell vacuolation, proliferation of bile ducts, lymphocyte infiltration, pericholangitis, and lymphocyte aggregate. The applied scores were multiplied by the parameters’ respective impact factors ranging from 1 to 3, which accounts for the effect of the alterations on the organ functionality. The microscopic ISI Total Score was obtained by summing up the product of these multiplications, where a higher number indicates a poorer health status of the assessed tissues.

#### Immunohistochemical Analysis

For the immunohistochemical analysis, ileum and liver samples collected in each age were fixed into zinc solution for at least 72 h. All the samples were dehydrated, infiltrated, and embedded into paraffin following common histological routine. The slides were then incubated with 500 µL of primary specific antibody for macrophages, CD4+, or CD8+ T Lymphocytes, followed by an incubation with HRP conjugated antibody specific for the primary antibody for 30 to 60 min. The peroxidase activity was blocked by using DAB kit for immunocytochemistry (HRP-conjugated rabbit anti-mouse Ig, Dako North America, Carpimteria, CA). The slides were then counterstained with hematoxylin solution. The labeled cells were counted under an optical microscope (400× magnification). Five fields per samples were evaluated.

#### Immune-Related Gene Expression

Ileum and liver samples collected on d 7, 14, 21, and 28 were stored in 1 mL tubes containing RNA later. Total RNA was isolated using Trizol reagent (Invitrogen, Carlsbad, CA) following manufacture's recommendations. Turbo-DNAse kit (Applied Biosystems, Foster City, CA) was sued to degrade any DNA present in the samples. RNA concentration was determined with NanoDrop Spectrophotometer (Thermo Scientific, Bonn, Germany) and the integrity by Experion Automated Electrophoresis System (Bio-Rad, Hercules, CA). One μg of mRNA was used to produce cDNA in a 20 µL reaction volume using iScript Reverse Transcription Supermix kit (Bio-Rad, Hercules, CA).

The final qPCR reaction was performed using the iTAq Universal SYBR Green Supermix (Bio-Rad, Hercules, CA). The cycle conditions for all primers used were as follow: initial denaturation step of 60 s at 95°C, followed by 40 cycles of annealing and extension of 30 s at 60°C. The melting profile of each sample was analyzed after every qPCR to confirm the product specificity. The results were analyzed the delta-delta equation and using an endogenous control (GAPDH; Table 2).

**Table 2:** Forward and reversed primer sequences used for qPCR analysis. Experiment 1.

Gene/Cytokine	Forward	Reverse
IL-10	CGGGAGCTGAGGGTGAA	GTGAAGAAGCGGTGACAGC
IL-8	CAGTTTCCTAGTCAGAGTCAGC	CCAAACCCACAGTCTTACAG
GAPDH	GGTGGTGCTAAGCGTGTTAT	ACCTCTGTCATCTCTCCACA

IL: interleukin; GAPDH: glyceraldehyde 3-phopshate dehydrogenase.

### *Statistical Analysis*

All the analyzed parameters were considered significantly different at *P* ≤ 0.05. Parametric data were analyzed by 1-way ANOVA and Tukey's test was used for pairwise comparison between groups (*P* < 0.05). Nonparametric data (ISI scores) were submitted to Wilcoxon’ test and the means separated by Dunn's test. The gene expression analysis was performed by t-test with Welch's correction at 10% probability.

### Experiment 2

The experiment was conducted at the research center Mercolab, Cascavel, Parana, Brazil. All the procedures followed the guidelines and were approved by the internal Animal Care Committee, and the activities followed by a certified veterinarian.

#### Animals, Experimental Design, and Diets

A total of 1,120 one-day old male Cobb 500 chicks were used in this trial. The birds were placed in floor pens with fresh litter in a completely randomized design. The experiment consisted of 4 treatment groups with 8 replicate pens per treatment and 35 birds per pen. The treatments were: Control (basal diet), basal diet plus supplementation of MUR at 25,000 LSU(F)/kg, basal diet plus supplementation of MUR at 45,000 LSU(F)/kg, and a positive control (basal diet plus Enramycin at 10 ppm during starter and grower and 5 ppm during the finisher phase). The birds had *ad libitum* access to water and feed during the entire experimental period that lasted 42 d. The MUR used in the present study is same used by Goes et al. (2022), and its activity and recovery rates are presented in Table 3.

**Table 3:** Muramidase activity (LSU (F)/kg) and recovery (%) from the feed in starter, grower, and finisher phases in Experiments 1 and 2.

Phase feeding, Exp. 1	MUR^1^ doses LSU (F)/kg		
	0	25,000	35,000
Starter (1–14 d)			
MUR activity LSU(F)/kg	-	18,189	32,635
MUR recovery (%)	-	72	93
Grower (14–28 d)			
MUR activity LSU(F)/kg	-	18,334	34,268
MUR recovery (%)	-	73	98
Phase feeding, Exp. 2	MUR^1^ doses LSU (F)/kg		
	0	25,000	45,000
Starter (1–21 d)			
MUR activity LSU(F)/kg	-	25,293	41,892
MUR recovery (%)	-	101	93
Grower (22–35 d)			
MUR activity LSU(F)/kg	-	21,479	34,246
MUR recovery (%)	-	86	76
Finisher (36–42 d)			
MUR activity LSU(F)/kg	-	24,744	48,591
MUR recovery (%)	-	99	108

1 Muramidase (gene coding muramidase 007 obtained from the fungus *Acremonium alcalophilum* (strain 114.92), Novozymes A/S (Bagsvaerd, Denmark) were included according to the treatments (0, 25,000 LSU (F)/kg, 35,000 LSU (F)/kg and 45,000 LSU (F)/kg).

The feed in mash form was produced at the feed mill located on Mercolab's research farm. The diets were formulated following commercial recommendations usually used in Brazil, based on corn and soybean meal to meet or exceed the requirements of the birds, according to each phase: Starter (0–21 d), grower (21–35 d), and finisher (35–42 d), with phytase (RONOZYME HiPhos GT) at 1,000 FYT/kg of feed. CAROPHLL yellow 10% at 40 ppm and monensin during starter and Salinomycin during the grower phase were also included in the basal feed as anticoccidials (Table 1).

#### Experimental Challenge

At 10, 11, and 12 d of the experiment all the birds were inoculated by oral gavage with a fresh broth culture of a field isolate *Clostridium perfringens* (0.5 mL containing 10^6^ CFU/bird).

#### Growth Performance and Carcass Yield

The birds and feed from each were weighed on d 21, 35 and 42 to calculate BWG, FI, and FCR. Mortality and weight of the dead birds were also recorded. At 42 d, the FCR was also corrected by a common BW (2.7 kg) to obtain cFCR. At 42 d, the birds from each pen were individually weighed to evaluate flock uniformity within each pen. The production efficiency index (**PEI**) was calculated on d 42. The PEI was calculated with the following formula: PEI = ((BW at d 42*(100 − mortality))/(42*FCR at d 42)*100).

At 42 d, 4 birds per replicate were used for carcass yield evaluation. Before slaughter, the birds were weighed, and the carcass (without feathers, head, feet, and viscera) and breast weight used to calculate carcass and breast yield, respectively.

#### Carotenoid Determination

At 21, 35, and 42 d, 2 birds per replicate were used to collect blood to measure total carotenoid concentration by using the iCheck CAROTENE device and using iEx CAROTENE reagent vials (BioAnalytGmbH, Teltow, Germany). The total carotenoid concentration is used as an indicator of intestinal integrity and absorption capacity (Rochell et al., 2016).

### *Statistical Analysis*

Data were checked for normality and presence of outliers (2 ± SD). Growth performance data, carcass yield, and total carotenoid concentration were analyzed by ANOVA (*P* < 0.05), and the means separated by Tukey's test. Additionally, in Experiment 2, the doses of MUR supplementation were submitted to polynomial regression analysis (linear and quadratic) to estimate the dose response by using JMP 16.0.

## RESULTS

### Experiment 1

#### Growth Performance

The growth performance results from 1 to 28 d are shown in Table 4. It was observed that the challenge significantly reduced the BWG of all challenged groups, regardless of the supplementation (*P* = 0.004). Feed intake was also affected by the treatment groups (*P* = 0.009) wherein the challenged birds supplemented with Enramycin showed lower FI than the nonchallenged control birds. Lastly, it was observed that the FCR was drastically impacted by the challenge (*P* = 0.02), but the supplementation of MUR at the 2 doses tested, and Enramycin partially counteracted this effect, wherein MUR improved the FCR by an average of 4.5 points compared to the challenged control birds.

**Table 4:** Growth performance from 1 to 28 d of age of broiler chickens according to the experimental groups. Experiment 1.

Treatment	BWG^1^, g	FI^2^, g	FCR^3^, g:g
NC^4^	1,858^a^	2,135^a^	1.150^b^
CC^5^	1,552^b^	2,059^ab^	1.333^a^
CC + MUR25^6^	1,567^b^	2,006^ab^	1.291^ab^
CC + MUR35^7^	1,570^b^	2,016^ab^	1.285^ab^
CC + ENR^8^	1,595^b^	1,955^b^	1.229^ab^
SEM	55.7	32.9	0.037
*P* value	0.004	0.009	0.02

1 Body weight gain.

2 Feed intake.

3 Feed conversion ratio.

4 Nonchallenged control.

5 Challenged control.

6 Muramidase at 25,000 LSU(F)/kg of feed.

7 Muramidase at 35,000 LSU(F)/kg of feed.

8 Enramycin at 10 ppm (SEM = standard error of mean; *n* = 7).

ab Mean with different letters in the same column are significantly different at *P* < 0.05 Tukey's test.

#### ISI Macroscopic Analysis

The results for the ISI macroscopic evaluation at different ages are presented in Tables 5 and 6. At 7 d, it was observed that the challenge led to an increased respiratory (*P* = 0.03) and internal (*P* = 0.009) scores, but the supplementation of MUR at 25,000 LSU(F)/kg of feed significantly reduced the respiratory score, and overall, MUR and Enramycin supplementation promoted an intermediary ISI Total Score (*P* = 0.03), although not different from the challenge control. At 14 d, the birds in the nonchallenged control group and the group supplemented with MUR at 35,000 LSU(F)/kg of feed presented a lower general intestinal score (*P* = 0.003), while the birds supplemented with Enramycin presented a higher score when compared to the nonchallenged group. Although not significant, MUR supplementation numerically reduced the total score (*P* = 0.02), while the group supplemented with Enramycin presented the worst score (Table 5). At 21 d, it was reported that MUR and Enramycin numerically attenuated the coccidiosis score although not different from the challenged control group, and supplementation of MUR at 35,000 LSU(F)/kg of feed and Enramycin numerically reduced the total score (*P* = 0.02). Lastly, at 28 d of age, the supplementation of MUR and Enramycin only numerically attenuated the general score of the birds (*P* = 0.03), the intestinal score (*P* = 0.04) and the total score (*P* = 0.05) without significant difference from the challenged control birds.

**Table 5:** Results for the ISI macroscopic evaluation of the whole bird conducted during necropsy at 7 and 14 d of chickens according to the experimental treatment groups. Experiment 1.

Treatment	General^1^	Respiratory^2^	Internal^3^	Intestine^4^	Coccidiosis^5^	Total score
	7 d					
NC^6^	0.42	1.28^ab^	0.85^b^	2.14	0.00	4.7^b^
CC^7^	0.14	2.57^a^	5.57^a^	6.57	2.57	17.4^a^
CC + MUR25^8^	0.28	0.00^b^	3.85^ab^	5.57	2.42	12.1^ab^
CC + MUR35^9^	0.42	0.85^ab^	4.42^ab^	6.28	2.00	14.0^ab^
CC + ENR^10^	0.28	2.14^ab^	5.71^a^	5.00	1.42	14.6^ab^
SEM	0.12	0.48	0.79	0.98	0.52	2.01
*P* value	0.77	0.03	0.009	0.17	0.07	0.03
	14 d					
NC^6^	0.42	0.00	1.14	2.28^b^	0.00	3.85^b^
CC^7^	0.71	1.71	3.14	4.57^ab^	0.57	10.71^ab^
CC + MUR25^8^	1.00	0.85	2.00	4.42^ab^	1.00	9.28^ab^
CC + MUR35^9^	0.28	0.00	2.71	2.00^b^	0.28	5.28^ab^
CC + ENR^10^	1.14	0.00	2.28	9.85^a^	0.57	13.85^a^
SEM	0.09	0.09	0.31	1.18	0.56	1.44
*P* value	0.29	0.06	0.58	0.003	0.61	0.02

1 External, appearance, muscle, and locomotor.

2 Trachea, heart, and air sac.

3 Proventriculus, gizzard, pancreas, kidney, liver, yolk/diverticulum, and thymus.

4 General score of duodenum, jejunum, ileum, and cecum.

5 Coccidiosis-related lesions in duodenum, Jejunum, ileum, and cecum.

6 Nonchallenged control.

7 Challenged control.

8 Muramidase at 25,000 LSU(F)/kg of feed.

9 Muramidase at 35,000 LSU(F)/kg of feed.

10 Enramycin (SEM = standard error of mean).

ab Mean with different letters in the same column are significantly different at *P* < 0.05 Dunn's test.

**Table 6:** Results for the ISI macroscopic evaluation of the whole bird conducted during necropsy at 21 and 28 d of chickens according to the experimental treatment groups. Experiment 1.

Treatment	General^1^	Respiratory^2^	Internal^3^	Intestine^4^	Coccidiosis^5^	Total score
	21 d					
NC^6^	0.14	0.28	0.14	4.14	0.00^b^	4.71^b^
CC^7^	0.14	0.00	0.85	10.26	3.28^a^	14.57^a^
CC + MUR25^8^	0.42	0.00	1.14	10.57	1.00^ab^	13.14^a^
CC + MUR35^9^	0.00	0.00	0.42	8.00	2.14^ab^	10.57^ab^
CC + ENR^10^	0.14	0.00	0.00	8.85	1.85^ab^	10.85^ab^
SEM	0.23	0.30	0.27	0.95	0.30	1.37
*P* value	0.06	0.41	0.33	0.06	0.02	0.02
	28 d					
NC^6^	0.14^b^	0.00	0.71	3.14^b^	0.00	4.00^b^
CC^7^	1.42^a^	0.42	1.00	8.00^ab^	1.00	11.85^a^
CC + MUR25^8^	0.42^ab^	0.00	0.85	9.42^a^	0.42	11.14^a^
CC + MUR35^9^	1.14^ab^	0.85	1.00	7.00^ab^	0.71	10.71^ab^
CC + ENR^10^	0.42^ab^	0.00	0.28	7.71^ab^	0.00	8.42^ab^
SEM	0.23	0.36	0.59	1.08	0.32	1.76
*P* value	0.03	0.54	0.53	0.04	0.19	0.05

1 External, appearance, muscle, and locomotor.

2 Trachea, heart, and air sac.

3 Proventriculus, gizzard, pancreas, kidney, liver, yolk/diverticulum, and thymus.

4 General score of duodenum, jejunum, ileum, and cecum.

5 Coccidiosis-related lesions in duodenum, jejunum, ileum, and cecum.

6 Nonchallenged control.

7 Challenged control.

8 Muramidase at 25,000 LSU(F)/kg of feed.

9 Muramidase at 35,000 LSU(F)/kg of feed.

10 Enramycin (SEM = standard error of mean).

ab Mean with different letters in the same column are significantly different at *P* < 0.05 Dunn's test.

### *ISI Histology Analysis*

#### Liver

The liver histopathology results are shown in Tables 7 and 8. At 7 d of age, it was observed that the supplementation of MUR at 35,000 LSU(F)/kg of feed significantly reduced the cell vacuolation score when compared to the challenged control group or the group supplemented with Enramycin (*P* < 0.001). This effect, added by nonsignificant reductions in other liver parameters, has reduced the ISI Total Score of challenged birds supplemented MUR at 35,000 LSU(F)/kg of feed to the same level of the nonchallenged animals (*P* < 0.001). At 14 d of age, supplementation of MUR at 25,000 LSU(F)/kg of feed reduced the score of congestion compared to the nonchallenged control group (*P* < 0.001), Enramycin reduced the proliferation of bile ducts (*P* = 0.002), and 25,000 LSU(F)/kg of feed of MUR and Enramycin numerically reduced the ISI Total Score (not different from the challenged control group). At 21 d, MUR at both doses and enramycin supplementation reduced the cell vacuolation (*P* < 0.001) and the ISI Total Scores (*P* < 0.001) compared to the challenged control group. At 28 d, birds supplemented with 35,000 LSU(F)/kg of feed of MUR and Enramycin showed reduced congestion score (*P* < 0.001), and numerical reduction in the ISI Total Score (*P* = 0.008) when compared to the challenged control birds.

**Table 7:** Results for the ISI histopathological evaluation of liver of chickens at 7 and 14 d according to the experimental treatment groups. Experiment 1.

Treatment	CON^1^	CV^2^	PBD^3^	LI^4^	PC^5^	LA^6^	Total score
	7 d						
NC^7^	0.20	0.40^d^	0.20	0.17	0.52	0.00	1.50^b^
CC^8^	0.38	2.70^a^	0.23	0.13	0.25	0.13	3.83^a^
CC + MUR25^9^	0.25	1.66abc	0.16	0.15	0.15	0.20	2.58^ab^
CC + MUR35^10^	0.23	1.50^c^	0.26	0.18	0.20	0.03	2.41^b^
CC + ENR^11^	0.30	2.33^ab^	0.20	0.18	0.40	0.16	3.58^a^
SEM	0.07	0.28	0.10	0.06	0.15	0.08	0.34
*P* value	0.31	<0.001	0.92	0.95	0.16	0.15	<0.001
	14 d						
NC^7^	0.32^b^	0.30^c^	0.25^ab^	0.15	0.45	0.05	1.52^b^
CC^8^	0.70^a^	0.50^bc^	0.54^a^	0.36	0.70	0.10	2.90^a^
CC + MUR25^9^	0.33^b^	0.90^ab^	0.50^a^	0.38	0.10	0.00	2.21^ab^
CC + MUR35^10^	0.65^ab^	1.33^a^	0.20^ab^	0.15	0.20	0.00	2.53^a^
CC + ENR^11^	0.65^ab^	0.33^c^	0.06^b^	0.36	0.35	0.10	1.86^ab^
SEM	0.10	0.15	0.12	0.08	0.21	0.06	0.31
*P* value	<0.001	<0.001	0.002	0.08	0.09	0.25	0.006

1 Congestion.

2 Cell vacuolation.

3 Proliferation of bile ducts.

4 Lymphocyte infiltration.

5 Pericholangitis.

6 Lymphocyte aggregate.

7 Nonchallenged control.

8 Challenged control.

9 Muramidase at 25,000 LSU(F)/kg of feed.

10 Muramidase at 35,000 LSU(F)/kg of feed.

11 Enramycin; (SEM = standard error of mean).

abcd Mean with different letters in the same column are significantly different at *P* < 0.05 Dunn's test.

**Table 8:** Results for the ISI histopathological evaluation of liver of chickens at 21 and 28 d according to the experimental treatment groups. Experiment 1.

Treatment	CON^1^	CV^2^	PBD^3^	LI^4^	PC^5^	LA^6^	Total score
	21 d						
NC^7^	0.35^b^	0.40^ab^	0.10	0.32	0.07	0.05	1.30^b^
CC^8^	0.68^ab^	0.73^a^	0.46	0.30	0.20	0.10	2.48^a^
CC + MUR25^9^	0.75^ab^	0.10^bc^	0.30	0.35	0.25	0.00	1.75^b^
CC + MUR35^10^	0.85^a^	0.06^bc^	0.26	0.31	0.55	0.00	2.05^b^
CC + ENR^11^	0.86^a^	0.00^c^	0.20	0.33	0.60	0.03	2.03^b^
SEM	0.11	0.11	0.12	0.08	0.21	0.04	0.29
*P* value	0.002	<0.001	0.14	0.98	0.45	0.21	<0.001
	28 d						
NC^7^	0.40^ab^	0.75^ab^	0.10^b^	0.10	0.15	0.00	1.50^b^
CC^8^	0.61^a^	0.56^b^	0.56^a^	0.35	0.80	0.03	2.93^a^
CC + MUR25^9^	0.50^ab^	0.66^b^	0.40^ab^	0.31	0.50	0.16	2.55^a^
CC + MUR35^10^	0.26^b^	1.20^a^	0.33^ab^	0.21	0.15	0.10	2.26^ab^
CC + ENR^11^	0.26^b^	0.96^ab^	0.30^ab^	0.26	0.35	0.03	2.18^ab^
SEM	0.08	0.16	0.12	0.07	0.25	0.07	0.35
*P* value	<0.001	0.003	0.05	0.06	0.07	0.44	0.008

1 Congestion.

2 Cell vacuolation.

3 Proliferation of bile ducts.

4 Lymphocyte infiltration.

5 Pericholangitis.

6 Lymphocyte aggregate.

7 Nonchallenged control.

8 Challenged control.

9 Muramidase at 25,000 LSU(F)/kg of feed.

10 Muramidase at 35,000 LSU(F)/kg of feed.

11 Enramycin; (SEM = standard error of mean).

ab Mean with different letters in the same column are significantly different at *P* < 0.05 Dunn's test.

#### Ileum

The ileal histopathology results are shown in Tables 9 and 10. At 7 and 14 d, there was an effect of treatments on all the scores evaluated (Table 9). Looking at the ISI Total Score, one could observe that the challenge increased the score without positive effect of the supplementation of MUR nor Enramycin. At 21 and 28 d (Table 10), effects of the treatments could be observed on all the evaluated parameters, except for congestion. At 21 d, the MUR supplementation at 35,000 LSU(F)/kg of feed for challenged birds reduced the lamina propria thickness compared to nonsupplemented challenged group (*P* < 0.001). Moreover, animals supplemented with 35,000 LSU(F)/kg of feed of MUR exhibited numerical decrease in the score of epithelial thickness, proliferation of enterocytes inflammatory, cell infiltration of lamina propria, and goblet cells in comparison to the challenged birds (*P* < 0.001; *P* = 0.002; *P* = 0.01, and *P* < 0.001, respectively), not significantly differing from the challenged control group.

**Table 9:** Results for the ISI histopathological evaluation of ileum of chickens at 7 and 14 d according to the experimental treatment groups. Experiment 1.

Treatment	LPT^1^	ET^2^		PE^3^	ICIE^4^	ICILP^5^	CON^6^	PO^7^	GC^8^	Total score
			7 d							
NC^9^	1.15^b^	0.72^b^		0.32^c^	0.00^d^	0.00^b^	0.25^c^	0.00^c^	1.10	3.55^c^
CC^10^	2.56^a^	1.35^a^		0.93^b^	0.11^cd^	0.05^b^	0.86^b^	3.60^b^	1.80	11.28^b^
CC + MUR25^11^	2.73^a^	1.58^a^		1.20^ab^	0.70^a^	0.70^a^	1.33^a^	4.10^b^	1.40	13.75^ab^
CC + MUR35^12^	2.56^a^	1.18^a^		1.26^ab^	0.35^bc^	0.05^b^	0.53^b^	4.40^ab^	1.46	11.81^b^
CC + ENR^13^	3.30^a^	1.41^a^		1.51^a^	0.38^b^	0.05^b^	0.10^c^	5.95^a^	2.10	14.81^a^
SEM	0.25	0.12		0.14	0.07	0.11	0.19	0.49	0.35	0.93
*P* value	<0.001	<0.001		<0.001	<0.001	<0.001	<0.001	<0.001	>0.05	<0.001
			14 d							
NC^9^	1.15^b^	0.60^b^		0.70^b^	0.10^b^	0.82^c^	0.85^ab^	0.00^b^	0.35^c^	4.57^b^
CC^10^	2.20^a^	0.96^ab^		1.00^ab^	0.43^a^	1.15^bc^	0.50^ab^	2.00^a^	1.26^ab^	9.56^a^
CC + MUR25^11^	2.40^a^	1.21^a^		1.20^a^	0.71^a^	2.25^a^	1.00^a^	2.45^a^	1.76^a^	13.00^a^
CC + MUR35^12^	2.73^a^	1.16^a^		1.10^ab^	0.66^a^	2.40^a^	0.43^b^	0.70^b^	1.86^a^	11.06^a^
CC + ENR^13^	2.20^a^	1.08^a^		1.00^ab^	0.45^a^	1.90^ab^	0.56^ab^	2.20^a^	1.03^bc^	10.43^a^
SEM	0.24	0.11		0.11	0.09	0.37	0.17	0.41	0.16	1.11
*P* value	<0.001	<0.001		0.01	<0.001	<0.001	0.03	<0.001	<0.001	<0.001

1 Lamina propria thickness.

2 Epithelial thickness.

3 Proliferation of enterocytes.

4 Inflammatory cell infiltration in the epithelium.

5 Inflammatory cell infiltration in the lamina propria.

6 Congestion.

7 Presence of oocysts.

8 Goblet cells.

9 Nonchallenged control.

10 Challenged control.

11 Muramidase at 25,000 LSU(F)/kg of feed.

12 Muramidase at 35,000 LSU(F)/kg of feed.

13 Enramycin (SEM = standard error of mean).

abcd Mean with different letters in the same column are significantly different at *P* < 0.05 Dunn's test.

**Table 10:** Results for the ISI histopathological evaluation of ileum of chickens at 21 and 28 d according to the experimental treatment groups. Experiment 1.

Treatment	LPT^1^	ET^2^		PE^3^	ICIE^4^	ICILP^5^	CON^6^	PO^7^	GC^8^	Total score
			21 d							
NC^9^	1.40^c^	0.72^c^		0.72^b^	0.50^b^	1.35^b^	0.55	0.00^b^	1.35^b^	6.35^b^
CC^10^	2.96^a^	1.11^ab^		1.10^a^	0.85^a^	2.85^a^	0.26	0.80^b^	1.26^ab^	11.21^a^
CC + MUR25^11^	2.26^ab^	1.16^a^		1.10^a^	0.86^a^	2.60^ab^	0.66	0.50^b^	1.93^a^	11.15^a^
CC + MUR35^12^	1.96^bc^	1.03abc		1.03^ab^	0.95^a^	2.40^ab^	0.66	0.75^b^	1.16^b^	9.81^a^
CC + ENR^13^	2.26^ab^	0.83^c^		0.81^ab^	0.51^b^	2.15^ab^	0.46	2.05^a^	0.43^c^	9.53^a^
SEM	0.21	0.09		0.09	0.09	0.34	0.16	0.29	0.18	0.83
*P* value	<0.001	<0.001		0.002	<0.001	0.01	0.06	<0.001	<0.001	<0.001
			28 d							
NC^9^	1.75^b^	0.62^b^		0.65^b^	0.32^b^	2.10	0.10	0.00^b^	0.30^b^	5.92^b^
CC^10^	2.66^a^	1.00^a^		1.03^a^	0.66^a^	3.35	0.30	0.55^a^	1.10^a^	10.66^a^
CC + MUR25^11^	2.36^ab^	1.08^a^		1.00^a^	0.75^a^	3.25	0.23	0.10^b^	1.53^a^	10.31^a^
CC + MUR35^12^	2.16^ab^	1.00^a^		0.95^ab^	0.78^a^	3.30	0.16	0.00^b^	1.06^ab^	9.43^a^
CC + ENR^13^	1.96^b^	0.93^ab^		0.88^ab^	0.93^a^	2.40	0.26	0.00^b^	1.46^a^	8.85^a^
SEM	0.21	0.09		0.09	0.09	0.36	0.11	0.10	0.19	0.77
*P* value	0.005	0.001		0.007	<0.001	0.06	0.56	<0.001	<0.001	<0.001

1 Lamina propria thickness.

2 Epithelial thickness.

3 Proliferation of enterocytes.

4 Inflammatory cell infiltration in the epithelium.

5 Inflammatory cell infiltration in the lamina propria.

6 Congestion.

7 Presence of oocysts.

8 Goblet cells.

9 Nonchallenged control.

10 Challenged control.

11 Muramidase at 25,000 LSU(F)/kg of feed.

12 Muramidase at 35,000 LSU(F)/kg of feed.

13 Enramycin (SEM = standard error of mean).

abcd Mean with different letters in the same column are significantly different at *P* < 0.05 Dunn's test.

It was observed that at 28 d, the supplementation of MUR and Enramycin reduced the presence of oocysts in the epithelium (*P* < 0.001), and animals supplemented with 35,000 LSU(F)/kg of feed of MUR exhibited numerical decrease of the goblet cell score (*P* < 0.001). Despite positive numerical improvement of 35,000 LSU(F)/kg of feed of MUR and Enramycin on the ileum ISI Total Score on both 21 and 28 d, none of the supplemented groups significantly differed from the challenged control group.

### *Immunohistochemistry Analysis*

#### Liver

**Figure 1:**
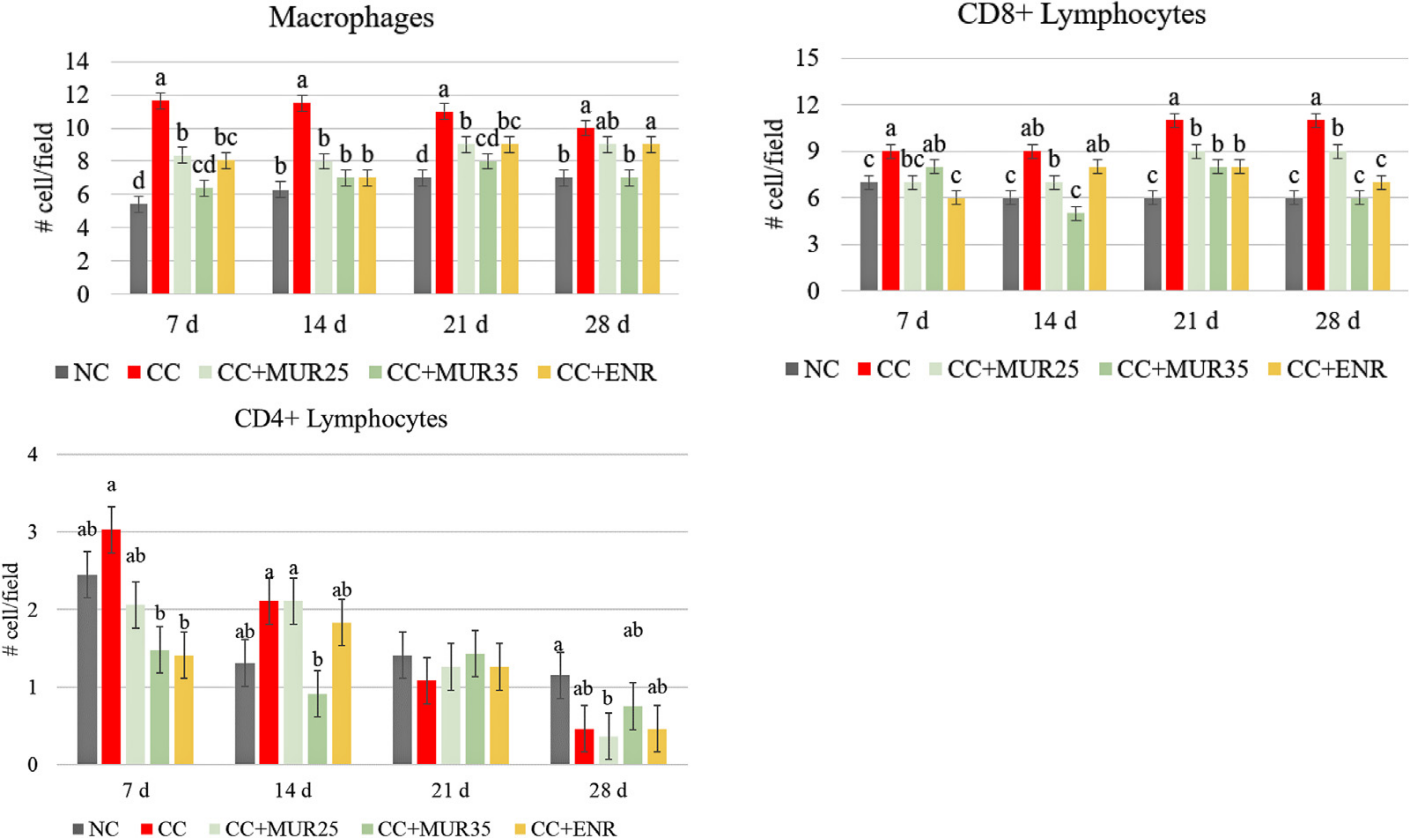
Immunohistochemistry results for macrophages, CD8+, and CD4+ cell (cells/field—400× magnification objective) in the liver of chickens at different ages according to the experimental treatment groups. Experiment 1. NC: nonchallenged control; CC: Challenged control; MUR25: Muramidase at 25,000 LSU(F)/kg of feed; MUR35: Muramidase at 35,000 LSU(F)/kg of feed; ENR: Enramycin. ^abc^Mean with different letters in the bars within the same age are significantly different at *P* < 0.05 Tukey's test.

The results presented in Figure 1 showed that the challenge significantly (*P* < 0.001) increased the infiltration of macrophages in the liver at all the ages. The supplementation of both doses of MUR and Enramycin significantly attenuated this effect on d 7, 14, and 21. However, the supplementation of 35,000 LSU(F)/kg of feed of MUR had a strong positive effect in reducing the macrophage infiltration in the liver to same level as the nonchallenged control birds at all the ages evaluated. Additionally, the challenge significantly increased the infiltration of CD8+ lymphocyte in the liver at all ages (*P* < 0.001). At 7 d, the supplementation of 25,000 LSU(F)/kg of feed of MUR reduced its infiltration to the same level as nonchallenged birds and as those supplemented with Enramycin. At 14 d, however, only the supplementation of 35,000 LSU(F)/kg of feed of MUR reduced the CD8+ lymphocyte infiltration to the same extent as the nonchallenged birds. At 21 d, all the supplemented groups reduced the CD8+ lymphocyte infiltration, and at 28 d, only the highest MUR dose and Enramycin reduced its infiltration to the same level as the nonchallenged group. Lastly, it was observed that at 7 d (first peak of coccidiosis cycling; *P* = 0.05) and 14 d (after the *C. perfringens* challenge; *P* = 0.006) the supplementation of 35,000 LSU(F)/kg of feed of MUR significantly reduced the CD4+ lymphocyte infiltration in the liver to the same level as nonchallenged birds, while Enramycin only reduced it at d 7. At 21 and 28 d, there was no clear separation between the groups and, in general, the infiltration was lower than at 7 and 14 d.

#### Ileum

**Figure 2:**
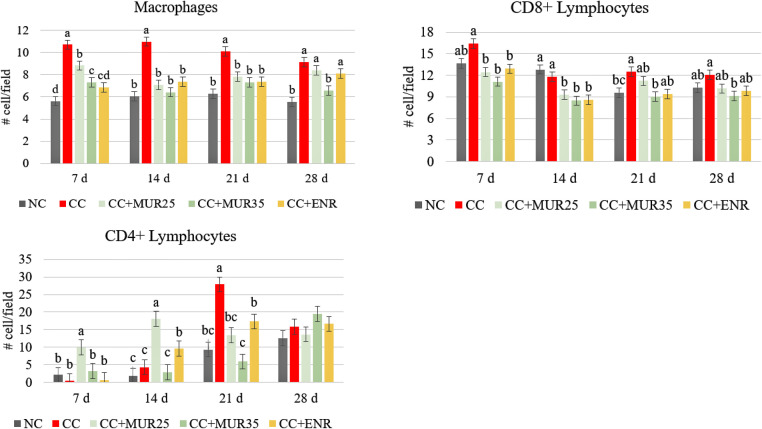
Immunohistochemistry results for macrophages, CD8+, and CD4+ cell (cells/field—400× magnification objective) in the ileum of chickens at different ages according to the experimental treatment groups. Experiment 1. NC: nonchallenged control; CC: Challenged control; MUR25: Muramidase at 25,000 LSU(F)/kg of feed; MUR35: Muramidase at 35,000 LSU(F)/kg of feed; ENR: Enramycin. ^abc^Mean with different letters in the bars within the same age are significantly different at *P* < 0.05 Tukey's test.

The results are presented in Figure 2. It was observed that the challenge significantly increased (*P* < 0.001) the infiltration of macrophages in the ileum at all the ages evaluated. At 7, 14, and 21 d both doses of supplementation of MUR reduced macrophage infiltration compared to the challenged control birds, but at 7 d the supplementation of 35,000 LSU(F)/kg of feed of MUR and Enramycin showed the greater effects. At 28 d, only the supplementation of 35,000 LSU(F)/kg of feed of MUR reduced the macrophage infiltration to the same level as the nonchallenged control birds, while the lower inclusion level of MUR and Enramycin supplementation did not differ from the challenge control group. Furthermore, at 7 and 14 d, both doses of MUR and Enramycin reduced (*P* < 0.001) the infiltration of CD8+ lymphocytes; however, at 21 (*P* < 0.001) and 28 d (*P* = 0.009), the supplementation of 35,000 LSU(F)/kg of feed of MUR had the largest effect in reducing the CD8+ lymphocyte infiltration. Lastly, at 7 and 14 d, the supplementation 25,000 LSU(F)/kg of feed of MUR promoted the highest infiltration of CD4+ lymphocyte when compared to the other treatments (*P* < 0.001). At 21 d, the challenged control birds showed the highest CD4+ lymphocyte infiltration in the ileum, followed by the birds supplemented with 25,000 LSU(F)/kg of feed of MUR and Enramycin (*P* < 0.001). No significant difference between the treatments was observed at 28 d of age.

### Expression of Immune-Related Genes

**Figure 3:**
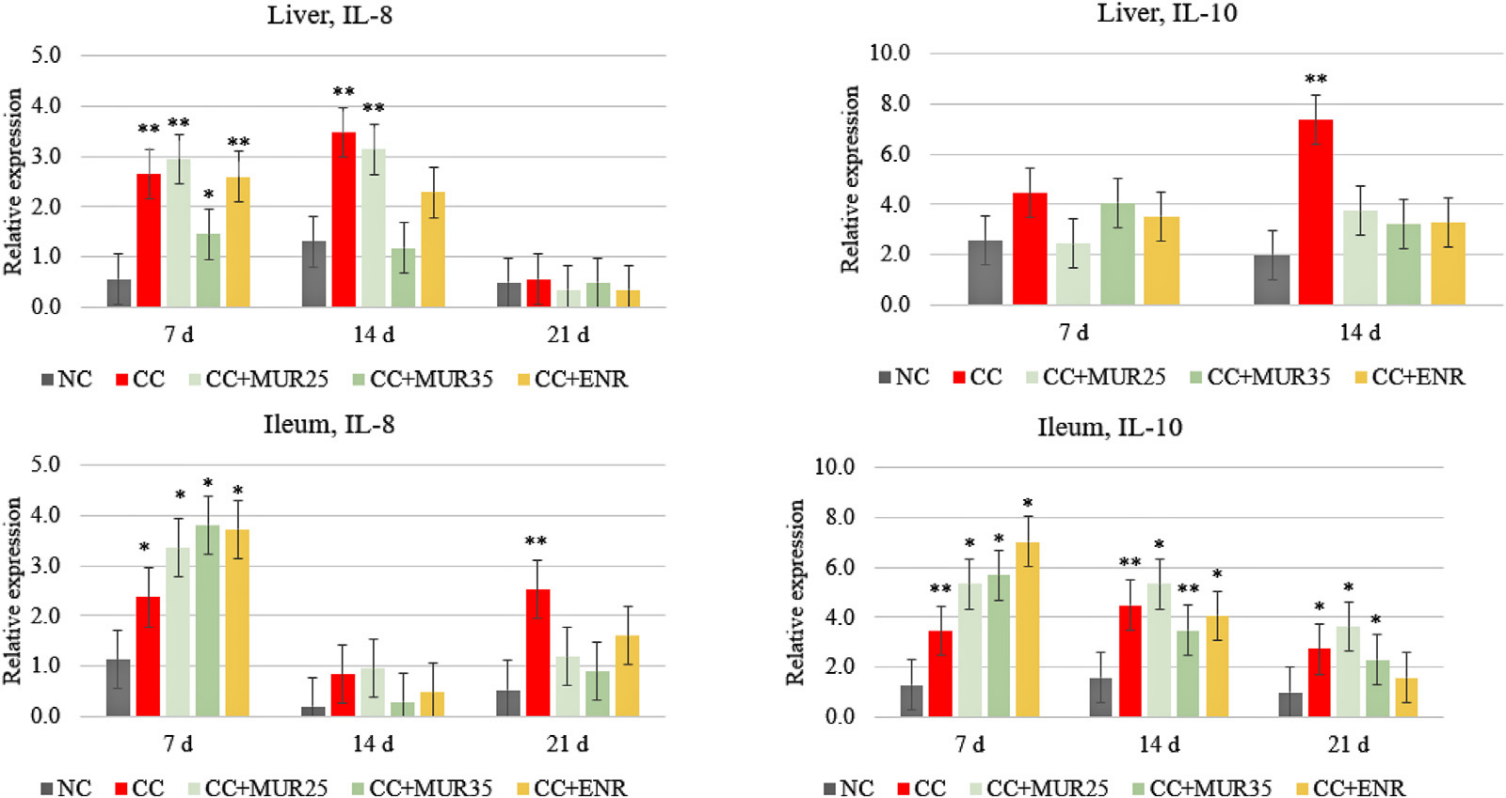
Relative mRNA expression of IL-8 and IL-10 in the liver and ileum of chickens at different ages according to the experimental treatment groups. Experiment 1. NC: nonchallenged control; CC: challenged control; MUR25: muramidase at 25,000 LSU(F)/kg of feed; MUR35: muramidase at 35,000 LSU(F)/kg of feed; ENR: enramycin. ^⁎⁎^*P* < 0.05 in the unpaired *t* test with Welch's correction when compared with the NC group; *P < 0.10 in the unpaired *t* test with Welch's correction when compared with the NC group.

The relative gene expression of IL-8 and IL-10 in the liver and ileum of chickens is shown in Figure 3. At 7 d, it was observed that the challenge upregulated the expression of IL-8 in the liver (*P* < 0.05), but the supplementation did not counteract this effect. However, at d 14, the supplementation of 35,000 LSU(F)/kg of feed of MUR and Enramycin prevented the upregulation of IL-8 in the liver which was not different from the nonchallenged control group. Additionally, the challenged upregulated the expression of IL-10 in the liver at d 14, but the supplementation of MUR at both doses and Enramycin counteracted this effect.

In the ileum, it was observed that the challenge upregulated the expression of IL-8 on d 7 and 21, and the supplementation of MUR at both doses and Enramycin counteracted this effect, but only at d 21. The challenge also increased the expression of IL-10 in the ileum at the 3 d evaluated, and only the supplementation of Enramycin, at d 21, reduced its expression to the same level as the nonchallenged control group.

### Experiment 2

#### Growth Performance, Carcass Yield, and Blood Carotenoids

The growth performance results of the Experiment 2 are shown in Table 11. It was observed that the BWG of the birds supplemented with MUR at both dosages was significantly improved, especially at the 1 to 35-day and 1 to 42-day periods (*P* < 0.05), when compared to the challenged control or supplemented with Enramycin, respectively. Considering the entire experimental period, from 1 to 42 d, a linear positive effect (*P* < 0.001) of the MUR doses was observed on the BWG. No significant differences were observed for FI. However, the body weight corrected FCR from 1 to 42 d was significantly improved by 5.6% with the supplementation of MUR (*P* = 0.02) when compared to the nonsupplemented birds. Additionally, a linear positive effect of MUR doses was observed on the FCR from 1 to 42 d (*P* = 0.008). The improved growth performance of MUR supplemented birds, also resulted in improved PEI when compared to the birds supplemented with Enramycin (*P* = 0.01); in addition, a linear positive effect of MUR doses was observed on the PEI at d 42 (*P* = 0.008). Viability and uniformity of the flock were not different between the treatment groups (*P* > 0.05).

**Table 11:** Growth performance results according to the experimental treatment groups. Experiment 2.

	Body weight gain, g			Feed intake, g			Feed conversion ratio, g:g					
Treatment	1–21 d	1–35 d	1–42 d	1–21 d	1–35 d	1–42 d	1–21 d	1–35 d	1–42 d^1^	Viability, %	PEI^2^	Uniformity, %
Control	979^b^	2,327^b^	2,987^ab^	1,258	3,422	4,623	1.287	1.511	1.555^a^	95.4	420^ab^	77.3
MUR^3^ 25,000	1,006^ab^	2,448^a^	3,078^a^	1,285	3,487	4,743	1.298	1.460	1.469^b^	96.8	455^a^	80.9
MUR^3^ 45,000	1,024^a^	2,448^a^	3,104^a^	1,263	3,458	4,730	1.271	1.475	1.468^b^	94.3	454^a^	74.9
Enramycin	988^ab^	2,368^ab^	2,914^b^	1,264	3,377	4,515	1.325	1.499	1.522^ab^	93.9	406^b^	69.1
SEM	11.0	28.5	50.7	19.9	51.0	79.1	0.0	0.0	0.0	1.4	13.0	3.4
*P* value ANOVA	0.01	0.002	0.04	0.82	0.51	0.18	0.12	0.10	0.02	0.48	0.01	0.13
Polynomial regression												
Linear	0.005^4^	<0.001	<0.001^6^	0.96	0.65	0.23	0.29	0.13	<0.001^7^	0.50	0.008^8^	0.64
Quadratic	0.82	0.03^5^	0.24	0.23	0.42	0.43	0.26	0.08	0.07	0.25	0.12	0.24

1 cFCR: body weight corrected FCR.

2 PEI: production efficiency index.

3 MUR: muramidase.

4 Equation: 979.44 + 0.10045*Dose *R*^2^ = 0.34.

5 Equation: 2,380.27 + 0.27118*Dose − 0.00107*(Dose − 223.91)^2^*R*^2^ = 0.59.

6 Equation: 2,994.95 + 0.26624*Dose *R*^2^ = 0.52.

7 Equation: 1.517 − 0.00020*Dose + 7.473e−7*(Dose − 222.73)^2^*R*^2^ = 0.52.

8 Equation: 424.66 + 0.08094*Dose *R*^2^ = 0.30 (SEM: standard error of mean; *n* = 8).

ab Mean with different letters in the same column are significantly different at *P* < 0.05.

The carcass yield and total blood carotenoid results are shown in Table 12. It was observed that both MUR doses evaluated increased breast yield when compared to the nonsupplemented birds (*P* = 0.05). The total concentration of carotenoids was increased within the supplementation of 45,000 LSU(F)/kg of feed of MUR and Enramycin when compared to the nonsupplemented group (*P* < 0.001). It was also observed a linear positive effect of MUR doses on the blood carotenoids concentration at d 42 (*P* = 0.006).

**Table 12:** Carcass and breast yield and total blood carotenoid concentration (mg/L) at 42 d of age.

Treatment	Carcass, %	Breast, %	Carotenoids, 21 d	Carotenoids, 35 d	Carotenoids, 42 d
Control	82.0	27.2^b^	2.83	4.03	4.46^c^
MUR^1^ 25,000	83.2	29.0^a^	3.07	4.27	4.88^bc^
MUR^1^ 45,000	83.4	29.0^a^	3.05	4.48	5.56^ab^
Enramycin	83.0	28.2^ab^	3.30	4.26	6.44^a^
SEM	0.46	0.48	0.24	0.24	0.33
*P* value ANOVA	0.13	0.05	0.62	0.67	<0.001
Polynomial regression					
Linear	0.08	0.06	0.58	0.25	0.006^2^
Quadratic	0.49	0.29	0.70	0.98	0.58

1 MUR: muramidase.

2 Equation: 4.4025413 + 0.0024241*Dose *R*^2^ = 0.16 (SEM: standard error of mean; *n* = 8).

abc Mean with different letters in the same column are significantly different at *P* < 0.05.

## DISCUSSION

In the studies presented herein we evaluated the impact of MUR supplementation in broiler chickens undergoing *Eimeria* and/or *Clostridium perfringens* challenge with focus on growth performance, histopathology of the liver and ileum, immune-related parameters, and blood carotenoids concentration. In Experiment 1, MUR supplementation had positive impact on several macro and microscopic aspects evaluated, and reduced the infiltration of inflammatory cells in both liver and ileum during all the ages evaluated and modulated the expression of immune-related genes in both organs. These results are of paramount importance to explain the anti-inflammatory effect of dietary MUR. In Experiment 2, targeting the evaluation of the growth performance of broilers, the immunoregulatory effect of MUR was translated into improved BWG (up to 95% of the breeder guidelines with MUR supplementation), FCR, breast yield, and absorption capacity, as measured by total blood concentration of carotenoids, especially at d 42 that allowed for the longest cumulative period of supplementation.

Regarding the macro and microscopic evaluations, it was observed that the severity of lesions observed herein agreed with previous published work by Belote et al. (2018) in both liver and ileum when using a similar challenge model; however, in the present study, we have also demonstrated that MUR reduced the severity of the lesions, especially in the liver. It is important to highlight that the supplementation of MUR reduced, at least partially, various macroscopic lesions evaluated, which most likely reflects the attenuated inflammatory response promoted by the enzyme, such as lower expression of IL-8, and lower inflammatory cell infiltration, which will be further discussed. The reduction in cell infiltration in the ileum and liver by MUR has not been previously reported, but it is an essential aspect to build a deeper knowledge around MUR supplementation for broilers undergoing an enteric challenge.

Several publications have shown the benefits in supplementing broiler chickens with the same MUR to improve the growth performance beyond their genetic potential (Sais et al., 2020; Pirgozliev et al., 2021; Brugaletta et al., 2022; Goes et al., 2022). Bortoluzzi et al. (2023) reported that MUR increased the BWG and improved the FCR of chickens. Additional effects observed were reduction of the intestinal viscosity and modulation in the diversity, composition, and predictive function of the ileal microbiota. These observations are probably indirect effects of the enzyme (degradation of PGN) in attenuating the inflammatory response, as shown in the present work, that may reduce goblet cells density, and select a more beneficial microbiota composition and function (Brugaletta et al., 2022; Bortoluzzi et al., 2023).

In the context of the present work, coccidiosis is ranked as the most important disease-related issue in broiler production (Blake et al., 2020), and is a well-known predisposing factor of necrotic enteritis, caused by *C. perfringens* (Prescott et al., 2016), due to the increased mucus production by the goblet cells following coccidiosis infection (Collier et al., 2008) as well as the leakage of plasma proteins into the intestinal lumen (Prescott et al., 2016). It has been demonstrated that T CD8+ lymphocytes, macrophages, and to a less extend CD4+ lymphocytes are the primary cells mediating the immune response against coccidia infection (Trout and Lillehoj, 1995). As the first line of immunological defense against infective agents, macrophages perform many functions, including phagocytosis and destruction of antigens, and secretion of cytokines that regulate the proliferation and maturation of other macrophages as well as lymphocytes (Qureshi, 1998).

Chicken macrophages respond and are activated by many foreign antigens, such as coccidia, that will lead to the production of essential nitric oxide metabolites (Qureshi, 1998) which plays import role in the parasite killing (Tan et al., 2014), as well as secretion of various proinflammatory mediators (He et al., 2011), that, if exacerbated, may lead to tissue damage. Macrophages can be activated by either Th1 cytokines, such as IFN-g, that will induce macrophages with more proinflammatory properties, or Th2 cytokines, such as IL-4 and IL-13, that will induce macrophages to display more anti-inflammatory and tissue repair characteristics (He et al., 2011). IL-10, on the other hand, has been described as a cytokine that inactivates macrophages by suppressing proinflammatory cytokines (O'Garra and Vieira, 2007). In Experiment 1 from the present study, the challenge increased macrophage recruitment in both liver and ileum at all 4 time points evaluated, but the supplementation of MUR, especially at the highest-level (35,000 LSU(F)/kg of feed), showed consistent effect in reducing macrophage infiltration. These results may explain the reduced damaged in the intestine by MUR supplementation as observed in some of the macroscopic evaluation parameters.

On the other hand, T cells are important key factors in driving the immune response against coccidiosis in chickens. As reviewed by Kim et al. (2019) the increased recruitment of T cells to the site of the infection induces the production of several proinflammatory cytokines with anticoccidial effects. On the other hand, it has been suggested that coccidia parasites have evolved to stimulate the production of IL-10 to inhibit the production of proinflammatory ones (Kim et al., 2019). We showed that the supplementation of MUR reduced the infiltration of CD8+ lymphocytes in both liver and ileum, and upregulated the expression of IL-10, in the ileum mainly, and downregulated IL-8, a proinflammatory cytokine, in both liver and ileum. According to Livaditi et al. (2006) IL-8 is an important biomarker in evaluating the intensity and mortality caused by sepsis, which may be because in mammals IL-8 specifically activates neutrophils and generation of reactive oxygen species. Similar studies have demonstrated that MUR upregulated the expression of NOD2 receptors, and reduced CD3+ T lymphocytes in the duodenal cell wall (Wang et al., 2021), and increased the concentration blood IL-10 in chickens (Amer et al., 2023). These results show that MUR have protective roles during the onset of the infection with anti-inflammatory and regulatory properties which leads to lower recruitment of inflammatory cells to the site of infection.

In conclusion, it was demonstrated that MUR supplementation elicited an anti-inflammatory response in birds undergoing a NE challenge model. The main observations sustaining this conclusion are the reduction in the infiltration of macrophages and CD8+ lymphocytes in the liver and ileum of infected birds and, thus, downregulation of IL-8 and upregulation of IL-10. These effects in attenuating the inflammatory response may explain the improved growth performance of supplemented birds (Experiment 2), and improved absorption capacity, as shown by the higher blood concentration of total carotenoids.

## References

[bib0001] Amer S.A., Farahat M., Gouda A., Abdel-Wareth A.A.A., Abdel-Warith A.-W.A., Younis E.M., Elshopakey G.E., Baher W.M., Saleh G.K., Davies S.J., Attia G.A. (2023). New insights into the effects of microbial muramidase addition in the diets of broiler chickens. Animals (Basel).

[bib0002] Belote B.L., Tujimoto-Silva A., Hümmelgen P.H., Sanches A.W.D., Wammes J.C.S., Hayashi R.M., Santin E. (2018). Histological parameters to evaluate intestinal health on broilers challenged with Eimeria and Clostridium perfringens with or without enramycin as growth promoter. Poult. Sci..

[bib0003] Blake D.P., Knox J., Dehaeck B., Huntington B., Rathinam T., Ravipati V., Ayoade S., Gilbert W., Adebambo A.O., Jatau I.D., Raman M., Parker D., Rushton J., Tomley F.M. (2020). Re-calculating the cost of coccidiosis in chickens. Vet. Res..

[bib0004] Bortoluzzi C., Perez-Calvo E., Segobola P., Bolsenm P.B., Sorbara J.O.B. (2023). Effect of microbial muramidase supplementation in diets formulated with different fiber profile for broiler chickens raised under various coccidiosis management programs. Poult. Sci..

[bib0005] Brugaletta G., De Cesare A., Laghi L., Manfreda G., Zampiga M., Oliveri C., Pérez-Calvo E., Litta G., Lolli S., Sirri F. (2022). A multi-omics approach to elucidate the mechanisms of action of a dietary muramidase administered to broiler chickens. Sci. Rep..

[bib0006] Cardoso Dal Pont G., Lee A., Bortoluzzi C., Farnell Y.Z., Gougoulias C., Kogut M.H. (2023). Novel model for chronic intestinal inflammation in chickens: (2) immunologic mechanism behind the inflammatory response. Dev. Compar. Immunol..

[bib0007] Collier C.T., Hofacre C.L., Payne A.M., Anderson D.B., Kaiser P., Mackie R.I., Gaskins H.R. (2008). Coccidia-induced mucogenesis promotes the onset of necrotic enteritis by supporting Clostridium perfringens growth. Vet. Immunol. Immunopathol..

[bib0008] Goes E.C., Dal Pont G.C., Maiorka A., Bittencourt L.C., Bortoluzzi C., Fascina V.B., Lopez-Ulibarri R., Calvo E.P., Beirão B.C.B., Caron L.F. (2022). Effects of a microbial muramidase on the growth performance, intestinal permeability, nutrient digestibility, and welfare of broiler chickens. Poult. Sci..

[bib0009] Goodarzi Boroojeni F., Männer K., Rieger J., Pérez Calvo E., Zentek J. (2019). Evaluation of a microbial muramidase supplementation on growth performance, apparent ileal digestibility, and intestinal histology of broiler chickens. Poult. Sci..

[bib0010] He H., Genovese K.J., Kogut M.H. (2011). Modulation of chicken macrophage effector function by TH1/TH2 cytokines. Cytokine.

[bib0011] Kim W.H., Chaudhari A.A., Lillehoj H.S. (2019). Involvement of T cell immunity in avian coccidiosis. Front. Immunol..

[bib0012] Kogut M.H., Genovese K.J., Swaggerty C.L., He H., Broom L. (2018.). Inflammatory phenotypes in the intestine of poultry: not all inflammation is created equal. Poult. Sci..

[bib0013] Kraieski A.L., Hayashi R.M., Sanches A., Almeida G.C., Santin E. (2017). Effect of aflatoxin experimental ingestion and Eimeira vaccine challenges on intestinal histopathology and immune cellular dynamic of broilers: applying an intestinal health index. Poult. Sci..

[bib0014] Livaditi O., Kotanidou A., Psarra A., Dimopoulou I., Sotiropoulou C., Augustatou K., Papasteriades C., Armaganidis A., Roussos C., Orfanos S.E., Douzinas E.E. (2006). Neutrophil CD64 expression and serum IL-8: sensitive early markers of severity and outcome in sepsis. Cytokine.

[bib0015] O'Garra A., Vieira P. (2007). TH1 cells control themselves by producing interleukin-10. Nat. Rev. Immunol..

[bib0016] Pérez-Calvo E., Aureli R., Sorbara J.O.B., Cowieson A.J. (2023). Dietary muramidase increases ileal amino acid digestibility of wheat and corn-based broiler diets without affecting endogenous amino acid losses. Poult. Sci..

[bib0017] Pirgozliev V., Simic A., Rose S.P., Pérez Calvo E. (2021). Dietary microbial muramidase improves feed efficiency, energy and nutrient availability and welfare of broilers fed commercial type diets containing exogenous enzymes. Br. Poult. Sci..

[bib0018] Prescott J.F., Parreira V.R., Mehdizadeh Gohari I., Lepp D., Gong J. (2016). The pathogenesis of necrotic enteritis in chickens: what we know and what we need to know: a review. Avian Pathol..

[bib0019] Qureshi M. (1998). Role of macrophages in avian health and disease. Poult. Sci..

[bib0020] Rochell S.J., Parsons C.M., Dilger R.N. (2016). Effects of *Eimeria acervulina* infection severity on growth performance, apparent ileal amino acid digestibility, and plasma concentrations of amino acids, carotenoids, and α1-acid glycoprotein in broilers. Poult. Sci..

[bib0021] Sais M., Barroeta A.C., López-Colom P., Nofrarías M., Majó N., Lopez-Ulibarri R., Calvo E.Pérez, Martín-Orúe S.M. (2020). Evaluation of dietary supplementation of a novel microbial muramidase on gastrointestinal functionality and growth performance in broiler chickens. Poult. Sci..

[bib0022] Sytwala S., Günther F., Melzig M.F. (2015). Lysozyme- and chitinase activity in latex bearing plants of genus Euphorbia—a contribution to plant defense mechanism. Plant Physiol. Biochem..

[bib0023] Tan J., Applegate T.J., Liu S., Guo Y., Eicher S.D. (2014). Supplemental dietary l-arginine attenuates intestinal mucosal disruption during a coccidial vaccine challenge in broiler chickens. Br. J. Nutr..

[bib0024] Traub S., von Aulock S., Hartung T., Hermann C. (2006). Invited review: MDP and other muropeptides — direct and synergistic effects on the immune system. J. Endotoxin Res..

[bib0025] Trout J.M., Lillehoj H.S. (1995). Eimeria acervulina infection: evidence for the involvement of CD8+ T lymphocytes in sporozoite transport and host protection. Poult. Sci..

[bib0026] Vidal M.-L., Gautron J., Nys Y. (2005). Development of an ELISA for quantifying lysozyme in hen egg white. J. Agric. Food Chem..

[bib0027] Wang Y., Goossens E., Eeckhaut V., Calvo E.P., Lopez-Ulibarri R., Eising I., Klausen M., Debunne N., Spiegeleer B.D., Ducatelle R., Immerseel F.V. (2021). Dietary muramidase degrades bacterial peptidoglycan to NOD-activating muramyl dipeptides and reduces duodenal inflammation in broiler chickens. Br. J. Nutr..

